# Clinical outcomes associated with speech, language and swallowing difficulties post-stroke

**DOI:** 10.4102/sajcd.v70i1.957

**Published:** 2023-10-10

**Authors:** Stephanie A. Kaylor, Shajila A. Singh

**Affiliations:** 1 Department of Communication Sciences and Disorders, Faculty of Health and Rehabilitation Sciences, University of Cape Town, Cape Town, South Africa; 2 Department of Health and Rehabilitation Sciences, Faculty of Health Sciences, University of Cape Town, Cape Town, South Africa

**Keywords:** stroke, acute care, prospective cohort, South Africa, private sector, speech therapy, dysphagia, outcomes

## Abstract

**Background:**

There is a lack of prospective research in South Africa’s speech therapy private sector, specifically, in the acute stroke population. There is a need to understand the quality of speech therapy services and outcomes post-stroke in the private sector.

**Objectives:**

This prospective cohort study investigated associations between speech, language, and swallowing conditions (i.e. dysarthria, apraxia of speech, aphasia, dysphagia), and outcomes post-stroke (i.e. length of hospital stay [LOS], degree of physical disability according to the Modified Rankin Scale [mRS], functional level of oral intake according to the Functional Oral Intake Scale [FOIS], dehydration, weight loss, aspiration pneumonia, mortality).

**Method:**

A prospective design was used to determine the incidence of speech, language, and swallowing conditions post-stroke. Convenience sampling was used to select participants (*N* = 68). Various statistical tests were used and the alpha level was set at Bonferroni correction *p* < 0.01.

**Results:**

Co-occurring speech, language, and swallowing conditions frequently occurred post-stroke (88%). Participants who were referred to speech therapy later than 24 h post-admission (52.94%) stayed in hospital for a median of 3 days longer than those who were referred within 24 h (*p* = 0.042). Dysphagia was significantly associated with moderate to severe physical disability (*p* < 0.01). Dysphagia with aspiration was significantly associated with poor functional level of oral intake, at admission and at discharge (*p* < 0.01). At discharge, aspiration pneumonia was significantly associated with severe physical disability (*p* < 0.01, *r* = 0.70).

**Conclusion:**

In South Africa’s private sector, co-occurring speech, language, and swallowing conditions commonly occurred post-stroke, and dysphagia was strongly associated with physical disability and poor functional level of oral intake. Length of hospital stay was increased by delayed speech therapy referrals.

**Contribution:**

This article contributes data on speech therapy services, communication and swallowing disorders post-stroke, and outcomes in South Africa’s private sector.

## Introduction

Stroke is a prevalent non-communicable disease worldwide and a leading cause of morbidity and mortality (Global Burden of Disease, 2019). In South Africa, stroke was the third leading cause of death (5.71% of total deaths) in 2019 (Institute for Health Metrics and Evaluation, 2019). In low- and middle-income countries (LMICs), such as South Africa, stroke morbidity and mortality rates have increased (Mayosi et al., 2009). A high prevalence of stroke incidence within South Africa places demand on the healthcare system (Maphumulo & Bhengu, 2019).

In South Africa, stroke is managed medically in public and private healthcare (Maphumulo & Bhengu, 2019). These two healthcare systems differ in funding, expenditure, accessibility, availability of resources, and service delivery (De Villiers, 2021; Young, 2016). Public hospitals serve the majority (84%) of South Africa’s population (Department of Health, 2011), whereas private hospitals serve the minority (16%) of the population who can afford medical aid insurance, which is expensive (Young, 2016). In the private sector, the patient demand is lower compared with the public sector and there is a large availability of human and technology resources (Pierpoint & Pillay, 2020; Young, 2016). South African private healthcare is presumably considered to be of high-quality, as it was ranked 6th overall in developed and developing nations, which is on par with developed countries such as Switzerland and Sweden (Burger & Christian, 2020). The high standard of South African private healthcare suggests high-quality service delivery and thus favourable outcomes; however, there is limited published information on stroke outcomes in the private sector.

The National Health Insurance (NHI) Proposal Scheme aims to manage South Africa’s health inequity and achieve universal quality health coverage and services for South African citizens, by transforming the relationship between public and private healthcare sectors (Pillay et al., 2020; South African Government, 2019; Van Niekerk et al., 2020). Understanding the quality of services and outcomes associated with South Africa’s private healthcare sector in comparison to the public sector, may be beneficial for improving health services and intervention, which can facilitate equitable and quality services for all individuals, including those with stroke (Burger & Christian, 2020; De Villiers, 2021).

Stroke can cause physical disability and speech, language, and swallowing difficulties (Langhorne et al., 2011), which should be managed immediately post-admission by a multi-disciplinary rehabilitation team (e.g. speech-language therapist [SLT] and physiotherapist), within the acute setting (NICE, 2010).

Speech, language and swallowing conditions (i.e. dysarthria, apraxia of speech, aphasia, dysphagia) commonly occur post-stroke (De Cock et al., 2020). A South African retrospective study in the public sector found individuals post-stroke, who were referred to an SLT, and who presented with speech, language, and swallowing conditions had an incidence of 395.7 (per 1000) (Balie et al., 2019). In isolation, dysarthria had the highest incidence (per 1000) post-stroke (91.4), followed by dysphagia (25.5), aphasia (23.4), and apraxia of speech (10.6) (Balie et al., 2019). An international prospective study (Stipancic et al., 2019) and a retrospective study (Flowers et al., 2013) found that dysphagia had the highest incidence post-stroke, respectively (32% [23, 41]; 44% [38, 51]), followed by dysarthria (26% [17, 35]; 42% [35, 48]) and aphasia (16% [9, 23]; 30% [25, 37]).

Stroke can result in poor physical and functional ability, which has an impact on activity and participation in daily events, such as eating independently. Associations between moderate to severe physical disability and aspiration pneumonia post-stroke have been reported in previous studies (Schwarz et al., 2018). Co-occurring aphasia and dysarthria post-stroke are also associated with increased physical dependence at discharge (Mitchell et al., 2021).

Dysphagia post-stroke is associated with poor functional level of oral intake, dehydration, malnutrition, and weight loss (Jeong et al., 2018). Individuals who have dysphagia post-stroke can have oral phase difficulties such as difficulty with chewing, reduced buccal and labial tone, bolus propulsion, and pharyngeal phase difficulties such as premature spillage and oral residue post-swallowing (Schimmel et al., 2017). Oral and pharyngeal phase difficulties post-stroke can lead to aspiration and influence a patient’s functional level of oral intake (i.e. oral versus non-oral feeding) as per the Functional Oral Intake Scale (FOIS). The FOIS is a 7-point ordinal scale used to describe an individual’s functional level of oral intake of food and liquid, with levels 1–3 describing varying degrees of non-oral intake, levels 4–7 describing varying degrees of oral intake, and levels 6–7 describing total oral intake of multiple consistencies with specific food limitations (FOIS = 6) or no restrictions (FOIS = 7) (Crary et al., 2005). Oral intake according to FOIS is associated with altered food and liquid consistencies as per the International Dysphagia Diet Standardisation Initiative (IDDSI), which is a framework consisting of eight levels (0–7) to describe food textures and liquid viscosity (IDDSI, 2019). Dysphagia and altered food and liquid consistencies can increase the risk for dehydration and weight loss (Jeong et al., 2018).

Dysphagia post-stroke can lead to aspiration and silent aspiration (Aoki et al., 2016). Dysphagia with aspiration is necessary but not sufficient to result in aspiration pneumonia (Martino et al., 2005). Additional risk factors may influence the development of aspiration pneumonia post-stroke, as listed in Table 1.

**TABLE 1:** Risk factors for aspiration pneumonia post-stroke.

Researchers (year)	Research design	Sample size and participant information	Risk factors
Aoki et al. (2016)	Prospective cohort, single-centre study	*N* = 305 Acute stroke; acute care hospital	Severe NIHSS score and lack of multidisciplinary stroke team management (SLT, Dietician, Physiotherapist, Occupational Therapist)
Dziewas et al. (2004)	Prospective cohort study	*N* = 100 Acute stroke; stroke unit	Low LOC, NGT feeding, poor functional outcome as described by mRS > 4, facial palsy
Finlayson et al. (2011)	Multicentre retrospective cohort study	*N* = 8251 participants with ischaemic stroke; regional stroke centres	Stroke severity, non-lacunar ischaemic stroke, older age, males, COPD, coronary artery disease, preadmission dependency
Feng et al. (2019)	Prospective cohort study	*N* = 1220 Acute stroke; acute care	Cerebral haemorrhagic stroke
Huang et al. (2006)	Prospective cohort study	*N* = 96 Participants with post-stroke dysphagia; tertiary hospital	Dependent for oral intake
Langdon et al. (2009)	Prospective cohort study	*N* = 330 ischaemic stroke; three large tertiary hospitals	Enteral nutrition
Murray and Scholten (2018)	Prospective cohort study	*N* = 100 inpatient rehabilitation facilities	Poor oral hygiene
Schwarz et al. (2018)	Retrospective cohort study	*N* = 110 ischaemic stroke; secondary hospital	NGT feeding
Sellars et al. (2007)	Prospective cohort study	*N* = 412 Acute stroke; acute care tertiary hospital	Cognitive impairment, age > 65 years, severe physical disability (mRS > 4), dysarthria, aphasia, abnormal water swallow test

Please see the full reference list of the article for more information.

NIHSS, National Institutes of Health Stroke scale; SLT, speech-language therapy; LOC, level of consciousness; NGT, nasogastric Tube; COPD, chronic obstructive pulmonary disease; mRS, Modified Rankin Scale.

Aspiration pneumonia post-stroke is more likely to occur if certain risk factors are present, such as poor oral hygiene, low level of consciousness (LOC), dependency for oral intake, and enteral nutrition (Kenzaka et al., 2017; Langmore et al., 1998; Mandell & Niederman, 2019; Murray & Scholten, 2018; Schwarz et al., 2018). Poor oral hygiene is associated with aspiration pneumonia because of changes and complications in the oral cavity post-stroke such as oral infections (Schimmel et al., 2017), being physically dependent for oral care (Murray & Scholten, 2018), and receiving enteral nutrition, which is because of bacterial colonisation from feeding tubes and infrequent oral care (Langdon et al., 2009; Langmore et al., 2002). Low LOC is associated with aspiration and aspiration pneumonia because of poor coughing reflex and difficulty coordinating breathing and swallowing (Kenzaka et al., 2017; Mandell & Niederman, 2019). Being dependent for oral intake is associated with aspiration pneumonia because of a lack of caregiver training on dysphagia post-stroke and how to feed individuals safely, as well as rushed feeding pace as a result of time constraints in the acute setting (Huang et al., 2006; Langmore et al., 1998, 2002; Wright et al., 2008). Enteral nutrition (e.g. Nasogastric Tubes [NGTs], Percutaneous Endoscopic Gastroscopy [PEG]) has also been associated with aspiration pneumonia post-stroke because of incorrect positioning when receiving enteral nutrition (i.e. supine or low Fowlers position), and limited mobilisation, which can also contribute to Gastro-Oesophageal Reflux (GOR) (Langdon et al., 2009; Mandell & Niederman, 2019). These four risk factors are an early warning for development of aspiration pneumonia and can be managed and prevented by the stroke multi-disciplinary teams in the acute setting (Aoki et al., 2016).

Dysphagia-related complications such as malnutrition, weight loss, dehydration (Jeong et al., 2018), and aspiration pneumonia (Schwarz et al., 2018) post-stroke have been associated with an increased risk for mortality (De Villiers et al., 2011; Feng et al., 2019), for up to 1-year post-stroke (Teh et al., 2018). Aphasia post-stroke has also been associated with increased risk for mortality (Lazar & Boheme, 2017; Lima et al., 2020). A retrospective study found individuals with aphasia post-stroke had higher mortality rates than those without aphasia (24.1% versus 10.7%, *p* = 0.004), which was associated with severe stroke (Lima et al., 2020).

The timeliness of SLT services in acute care can impact an individual’s length of hospital stay (LOS) (Viljoen et al., 2014). Delayed SLT referrals (i.e. > 24 h) and speech, language, and swallow screening post-stroke are associated with delayed SLT intervention and subsequent consequences such as aspiration and aspiration pneumonia (Bray et al., 2017; Han et al., 2018; Viljoen et al., 2014). Preventable consequences in acute care are associated with unfavourable outcomes such as an increased LOS (Viljoen et al., 2014).

Early SLT assessment and intervention can assist with management of speech, language and swallowing conditions and prevention of adverse outcomes (Bernhardt et al., 2017). A high frequency of communication and swallowing intervention over short-term periods in acute care can assist with quicker recovery of speech, language, and swallowing functioning (Harvey et al., 2021).

Research on speech, language, and swallowing conditions post-stroke in the acute setting is largely based on international research and South Africa’s public sector (Balie et al., 2019; De Villiers et al., 2011; Viljoen et al., 2014). Limited information in the private sector makes it difficult to make comparisons on stroke outcomes between the two healthcare sectors. There is a need to understand the quality of SLT services provided to manage speech, language, and swallowing conditions, and outcomes post-stroke and if there is a need to improve acute care stroke services in the private sector.

The aims of this study were to determine the associations between speech, language, and swallowing conditions post-stroke and clinical outcomes (i.e. length of stay [LOS], degree of physical disability [Modified Rankin Scale {mRS 0–6}], functional level of oral intake [Functional Oral Intake Scale {FOIS 1–7}], dehydration, weight loss, and mortality); and to determine the associations between dysphagia, aspiration, aspiration pneumonia, and clinical outcomes. The objectives of this study were to (1) determine the incidence of dysarthria, apraxia of speech, aphasia, dysphagia, aspiration, and aspiration pneumonia post-stroke, (2) describe the nature of speech therapy services and intervention, and how the timeliness and nature of services influence stroke outcomes, and (3) determine the associations between risk factors (i.e. poor oral hygiene, dependency for oral intake, low LOC, enteral nutrition), and aspiration pneumonia.

## Research methods

### Design and setting

The researcher conducted a prospective and multisite study design between February and November 2020, to observe a cohort of adults (i.e. > 18 years) who presented with speech, language, and swallowing difficulties post-stroke. The study was set at five hospitals, representing four private hospital network groups in the Cape Metropolitan area.

### Study population

The study included all adults who were admitted for a new incident of acute stroke and who presented with speech, language and swallowing difficulties. The study excluded individuals with pre-existing speech, language, and swallowing difficulties.

### Sampling strategy

A power analysis calculation yielding *N* = 85 was required for the study, as verified on the advice of a biostatistician. The advent of coronavirus disease 2019 (COVID-19) pandemic had an impact on lower number of stroke admissions (Rinkel et al., 2020) and thus less speech therapy referrals. The total number of participants in this study was *N* = 68. Convenience sampling was used to select participants. Participants were selected at the respective hospital sites if they met the inclusion criteria of the study. All eligible individuals who consented to participate in the study were included.

### Participants

The median age of participants was 70.5 years (interquartile range [IQR]: 60–79). Most participants spoke English and Afrikaans (51.5%), which was likely because of the geographical location of the research. Most participants were retired (57.35%). The majority of participants had hypertension (80.88%). Ischaemic stroke was the most prevalent type of stroke in this study (75%).

### Data collection and procedures

The research personnel involved in data collection were four SLTs, including the researcher, who were registered with the Health Professions Council of South Africa (HPCSA), and who provided speech therapy services as part of standard care, at the private hospital sites. Informed consent was obtained from all participants, either by the participant or via proxy consent in the case of those who had language or cognitive challenges that restricted them from making an informed decision.

Two outcome measure scales were administered by the research personnel at admission and discharge, namely the FOIS (Crary et al., 2005), and the mRS (Banks & Marotta, 2007). The FOIS (Crary et al., 2005) (Appendix 1) has high inter-rater reliability with perfect agreement on 85% of ratings (Kappa statistics ranged from 0.86 to 0.91) (Crary et al., 2005). The mRS (Appendix 2) is a 6-point scale designed to measure the degree of physical disability for individuals post-stroke, from 0 = no symptoms at all, to 6 = death (Banks & Marotta, 2007). The mRS has moderate inter-rater reliability (kappa = 0.56) and strong test-retest reliability (kappa = 0.81 to 0.95) (Banks & Marotta, 2007). The treating SLT consulted with the participant’s physiotherapist about the mRS score.

Data were collected on demographic details, stroke details, pre-existing comorbidities, admission and discharge information, dentition, medication, SLT diagnosis, dysphagia assessment and dysphagia-related outcomes, and nature of SLT services and intervention. The research personnel recorded these details in participants’ medical folders, and this information was also uploaded to an electronic storage system. The researcher accessed participant data from the electronic system on a weekly basis. Data were abstracted onto an electronic data abstraction tool (Gregory & Radovinsky, 2012), which was created using Microsoft Excel and was based on the tool used by Balie et al. (2019). A coding manual was created to guide data abstraction (Appendix 3). The coding manual contained a key to describe the codes used, which represented specific variables and explained how to enter data to ensure that participant information was abstracted objectively and uniformly (Gregory & Radovinsky, 2012).

### Validity and reliability

Internal validity was achieved by controlling for confounding variables by means of excluding participants with pre-existing speech, language and swallowing difficulties as a result of a previous stroke, degenerative conditions, and COVID-19. Information from participants’ hospital folders were collected in accordance with the aims and objectives of the study, and data were rechecked, which ensured that the relevant content was abstracted (Gregory & Radovinsky, 2012). Descriptions for each severity rating of the scales were discussed with the research personnel to gain consensus and to avoid inconsistent scoring (Gregory & Radovinsky, 2012). To increase validity and reliability, a pilot study was conducted on three individuals post-stroke prior to the main study to test the feasibility of procedures and recruitment and consent rate, to pilot the outcome measure scales, to identify errors in the protocol, and to assess the need for further training of the SLTs collecting and abstracting the data (Hassan et al., 2006). Errors in entering data onto the abstraction tool were identified and changes were made to the study protocol. Intra-rater reliability was achieved by the researcher and second data abstractor for re-checking their own data entries, blind to the original data set, to ensure that all relevant information was captured consistently (Hallgren, 2012). Inter-rater reliability was assessed by the researcher and second data abstractor abstracting data for the first 10 participants, blind to the original data set (Scheel et al., 2018). The percentage of agreement was calculated using Cohen’s Kappa coefficient to assess inter-rater reliability (Hallgren, 2012). There was perfect agreement (1.00) for seven variables and near perfect agreement (0.81–0.99) for 10 variables. Inconsistent data entries were identified and managed by reviewing participants’ hospital folders and SLT consultation notes, and making corrections to the data entries.

### Data analysis

Data were cleaned by rechecking data entries, and missing data were managed by reviewing participants’ hospital folders and SLT consultation notes to obtain the relevant data. If missing data could not be obtained, these variables were excluded from statistical analyses for the specific case. Data from the abstraction tool was analysed by IBM^®^ SPSS^®^ 26. The alpha level was set at Bonferroni correction *p* < 0.01. Various statistical measures were used to analyse the data. A series of Mann–Whitney *U* tests were used to determine the associations between speech, language and swallowing conditions and timeliness of SLT referral, number of intervention sessions, and clinical outcomes (i.e. LOS, degree of physical disability [mRS values], functional level of oral intake [FOIS values]). Mann–Whitney *U* tests were also used to determine the associations between aspiration, aspiration pneumonia and clinical outcomes. Pearson’s chi-square tests of independence (Cramer’s *V*) or Fischer’s exact test was used to determine the proportion of participants with dehydration and mortality, the association between the four risk factors (i.e. low LOC, poor oral hygiene, enteral nutrition, and dependent for oral intake) and aspiration pneumonia, and the associations between aspiration, aspiration pneumonia and clinical outcomes (i.e. mortality, dehydration, weight loss). An odds ratio (OR) was calculated to determine the likelihood of mortality post-stroke for dysphagia, aphasia, dysarthria, and apraxia of speech.

### Ethical considerations

Ethics approval was obtained in November 2019 from the University of Cape Town (UCT), Faculty of Health Sciences (FHS), Human Research Ethics Committee (HREC/730-2019), prior to the commencement of the study. Permission to conduct the study was obtained from the Chief Executive Officers (CEOs) of the five acute care private hospitals. The study was conducted according to Helsinki 2013 guidelines (World Medical Association, 2013). Participants had the freedom to withdraw from the study at any point. Participant data that were uploaded onto the electronic storage system was secured by an authentication process using an email address and password to maintain patient confidentiality. The study’s dataset had no identifying information. A coding system was used where each participant was assigned a respective code for de-identification. A separate password-protected electronic document was created with the participants’ names and their respective codes to ensure that reliability checks could be conducted. There was no participant coercion in enrolling participants into the study.

## Results

### Speech, language and swallowing conditions

Of the 68 participants who were referred to SLT post-stroke, 53 (78%) participants presented with dysphagia, 50 (73.53%) presented with aphasia, 50 (73.53%) presented with dysarthria, and 11 (16.17%) presented with apraxia of speech. The majority of participants (88%) had co-occurring diagnoses. Aphasia, dysarthria, and dysphagia were the most commonly co-occurring diagnoses (38.23%) (Table 2).

**TABLE 2:** Speech, language and swallowing conditions, (*N* = 68).

Variable	*n*	%
**Conditions in isolation**		
Dysarthria	3	4.41
Aphasia	3	4.41
Dysphagia	2	2.94
Apraxia of speech	0	0.00
**Co-occurring conditions**		
Aphasia, dysarthria, dysphagia	26	38.23
Dysarthria, dysphagia	10	14.70
Aphasia, dysphagia	7	10.30
Aphasia, dysarthria	6	8.82
Aphasia, apraxia	3	4.41
Apraxia, dysarthria, dysphagia	3	4.41
Aphasia, apraxia, dysphagia	3	4.41
Aphasia, dysarthria, apraxia, dysphagia	2	2.94

### Nature of services

#### Referrals

All 68 (100%) participants were referred by their physicians to SLT services. The median time from admission to SLT referral (hours) was 48 h for the four speech, language and swallowing conditions. There were 32 (47.06%) participants who were referred to speech therapy within 24 h. Participants who were referred later than 24 h stayed in hospital for a median of 3 days longer than those who were referred within 24 h (*p* = 0.042) (Table 3).

**TABLE 3:** Association between speech-language therapy referral time and length of hospital stay.

SLT referral time and length of stay	Early (within 24 h) (*n* = 32)		Late (> 24 h) (*n* = 36)		*W*	*p*	Effect size
	Median	IQR	Median	IQR			
Length of hospital stay	12	7–17.5	15	11.5–21.5	398	0.042	0.29

Note: *p* < 0.01 at Bonferroni adjusted alpha level.

SLT, speech-language therapy; IQR, interquartile range.

#### Assessment

The majority of all the participants (89.70%) had Clinical Swallow Evaluations (CSE). Instrumental measures were not used as frequently: Fibreoptic endoscopic evaluation of swallowing (FEES) (1.47%), videofluroscopy (VFSS) (5.88%), which may have been because of difficulty in transferring patients with physical disability (Schwarz et al., 2018) and accessibility challenges because of COVID-19 (Miles et al., 2021). Participants’ treating SLT assessed their speech and language abilities using varied informal and formal assessment tools (e.g. Mount Wilga High Level Language Assessment).

#### Intervention sessions

Participants received an average of 11 intervention sessions in a median of 15 days. Participants with apraxia of speech had a median of four more intervention sessions compared with those without apraxia, whereas participants with aphasia had 3.5 more intervention sessions compared with those without aphasia.

#### Types of intervention

Participants with dysphagia (78%) received different types of intervention including compensatory strategies (e.g. modification to oral intake), swallow exercises (e.g. effortful swallows), and sensory stimulation (thermal tactile stimulation). All 53 participants with dysphagia received compensatory strategies (100%); most participants received swallow exercises (54.71%) and some participants received sensory stimulation (38.85%). The majority of participants who had aphasia received expressive language intervention (86%), and therapy for word-retrieval, including semantic feature analysis (76%). The majority of participants who had dysarthria received intervention for articulation of whom 54% had consonant exaggeration. Therapy for prosody, such as rate modification, was a prominent form of dysarthria management (46%). Eight participants who had apraxia of speech received therapy for automatic speech (72.72%), and articulatory kinematic therapy (72.72%). Four participants who had aphasia (8%) and four participants who had apraxia of speech (36.36%) received augmentative and alternative communication (AAC) intervention.

### Associations between clinical outcomes and speech, language, and swallowing conditions

#### Length of stay

The median LOS across all four conditions (i.e. dysphagia, aphasia, dysarthria, apraxia of speech) was 15 days (IQR: 9–20). Participants with dysphagia stayed in hospital for a median of 6 days longer than those without dysphagia (*p* = 0.022). Similarly, participants with aphasia had a median of 4.5 days longer hospital stay than those without aphasia (*p* = 0.023). Although not significant, participants with dysphagia who aspirated stayed for a median of 3 days longer in hospital compared with those who did not aspirate (*p* = 0.091), and participants who developed aspiration pneumonia stayed for a median of 5 days longer in hospital compared with those without aspiration pneumonia (*p* = 0.120). There were 29 participants (42.64%) who were discharged to rehabilitation or step-down facilities; 29 participants (42.64%) who were discharged home; and 10 participants (14.70%) died.

#### Level of physical disability

At admission and discharge, dysphagia was significantly associated with moderate to severe physical disability (mRS: 3–5) (*p* < 0.01) (Table 4). At discharge, dysphagia with aspiration pneumonia was significantly associated with severe physical disability (*p* < 0.01) (*n* = 5; 100%), with a strong effect size (*r* = 0.70) (Table 5). Most participants with co-occurring speech, language and swallowing conditions had moderate to severe physical disability (mRS: 3–5) at discharge.

**TABLE 4:** Association between speech, language, and swallowing conditions and degree of physical disability (*N* = 68).

Total participants	*n*	Median	IQR	*W*	*p*	Effect size
Dysphagia						
Admission mRS	-	-	-	127.5	< 0.001*	0.68
Discharge mRS	-	-	-	201.5	0.003*	0.49
Dysphagia	53	-	-	-	-	-
Admission mRS	-	4	4–5	-	-	-
Discharge mRS	-	4	3–5	-	-	-
No Dysphagia	15	-	-	-	-	-
Admission mRS	-	2	1–4	-	-	-
Discharge mRS	-	1	1–4	-	-	-
**Aphasia**						
Admission mRS	-	-	-	454.5	0.953	0.01
Discharge mRS	-	-	-	446	0.96	0.01
Aphasia	11	-	-	-	-	-
Admission mRS	-	4	3.25–5	-	-	-
Discharge mRS	-	4	2-5	-	-	-
No Aphasia	57	-	-	-	-	-
Admission mRS	-	4	3.25–5	-	-	-
Discharge mRS	-	4	2.25–4	-	-	-
**Dysarthria**						
Admission mRS	-	-	-	490	0.562	0.09
Discharge mRS	-	-	-	554.5	0.138	0.23
Dysarthria	50	-	-	-	-	-
Admission mRS	-	4	3–5	-	-	-
Discharge mRS	-	4	2–4	-	-	-
No dysarthria	18	-	-	-	-	-
Admission mRS	-	4	4–5	-	-	-
Discharge mRS	-	4	4–5	-	-	-
**Apraxia**						
Admission mRS	-	-	-	297	0.779	0.05
Discharge mRS	-	-	-	298	0.797	0.05
Apraxia	11	-	-	-	-	-
Admission mRS	-	4	3.5–5	-	-	-
Discharge mRS	-	4	3.5–4.5	-	-	-
No Apraxia	57	-	-	-	-	-
Admission mRS	-	4	3–5	-	-	-
Discharge mRS	-	4	2–5	-	-	-

mRS, Modified Rankin Scale; IQR, interquartile range.

* , *p* < 0.01 at Bonferroni adjusted alpha level.

**TABLE 5:** Association between aspiration pneumonia and degree of physical disability.

Degree of physical disability	Dysphagia (*n* = 53)				*W*	*p*	Effect size
	With aspiration pneumonia (*n* = 5)		Without aspiration pneumonia (*n* = 48)				
	Median	IQR	Median	IQR			
**Clinical indicators**							
Admission mRS	5	5–5	4	4–5	83	0.227	0.31
Discharge mRS	5	5–6	4	3–4.25	35.5	0.008*	0.70

mRS, Modified Rankin Scale; IQR, interquartile range.

* , *p* < 0.01 at Bonferroni adjusted alpha level.

#### Functional level of oral intake

At admission and discharge, dysphagia with aspiration was strongly associated with poor functional level of oral intake (i.e. altered consistency diets [FOIS: 4–6] and enteral nutrition [FOIS: 1–3]) (*p* < 0.01) (Table 6).

**TABLE 6:** Association between dysphagia with aspiration and functional level of oral intake.

Clinical indicators	Dysphagia (*n* = 53)				*W*	*p*	Effect size
	With aspiration (*n* = 34)		Without aspiration (*n* = 19)				
	Median	IQR	Median	IQR			
Admission FOIS	5	1–5	6	5–6	485.5	0.002*	0.50
Discharge FOIS	5	1–6	7	6–7	479.5	0.003*	0.49

FOIS, Functional Oral Intake Scale; IQR, interquartile range.

* , *p* < 0.01 at Bonferroni adjusted alpha level.

There was not a significantly greater number of participants who were on oral intake at discharge (*n* = 53) relative to admission (*n* = 52). Most participants with co-occurring speech, language and swallowing conditions had FOIS < 7 scores at discharge, indicating a lower level of oral intake and enteral nutrition. There were 16 (30.18%) participants who were placed on enteral nutrition following admission, and 15 (28.30%) participants were receiving enteral nutrition at discharge. At discharge, 27 participants (50.94%) improved their functional level of oral intake, nine participants (16.98%) remained on the same altered consistency diet, and seven participants (13.20%) had a lower level of functional oral intake (i.e. altered consistency diet or enteral nutrition) (Table 7). The majority of participants who developed aspiration pneumonia had enteral nutrition at discharge (median FOIS = 1) (*n* = 4; 80%) (*p* = 0.019).

**TABLE 7:** Mode of nutritional intake at admission and discharge.

Mode of nutritional intake	Admission		Discharge	*n*	%
	*n*	%			
**Altered consistency diets**					
Puree (IDDSI 4)	14	26.41	NPO 2 (NGT) died in hospital	2	14.28
			Puree (IDDSI 4)	1	7.14
			Soft (IDDSI 6)	7	50.00
			Regular (IDDSI 7)	4	28.57
Soft (IDDSI 6)	23	43.39	NPO 1 (NGT) discharged to rehab 2 (NGT) died in hospital	3	13.04
			Puree (IDDSI 4)	2	8.70
			Soft (IDDSI 6)	8	34.78
			Regular (IDDSI 7)	10	43.48
Regular (IDDSI 7)	15	28.30	Regular (IDDSI 7)	15	28.30
**Enteral nutrition**					
NPO	16	30.18	NPO 1 (NGT) went home 3 (NGT) discharged to rehab 4 (NGT) died in hospital 1 (PEG) went home 1 (PEG) died in hospital	10	62.50
			Puree (IDDSI 4)	4	25.00
			Soft (IDDSI 6)	1	6.25
			Regular (IDDSI 7)	1	6.25

IDDSI, International Dysphagia Diet Standardisation Initiative; NGT, nasogastric tube; PEG, percutaneous endoscopic gastroscopy; NPO, nil per Os (nil per mouth).

#### Dehydration

There were three participants (4.41%) who presented with dehydration on admission. All three participants presented with dysphagia and co-occurring speech and language conditions. Dehydration on admission was not significantly associated with any of the presenting speech, language and swallowing conditions. Two (66.66%) of these participants with dehydration on admission died prior to discharge.

#### Mortality

There were 10 (14.70%) participants who died during the study period. Participants who died had two to four co-occurring speech, language and swallowing conditions, of whom nine (90%) had dysphagia. Most participants who died had co-occurring aphasia, dysarthria and dysphagia (30%), and co-occurring dysarthria and dysphagia (30%). Participants with dysphagia had a higher odds (2.86) of dying compared with those without dysphagia. Mortality was not significantly associated with aspiration nor with aspiration pneumonia.

### Association between aspiration pneumonia and risk factors

There were 53 participants (78%) who had dysphagia, of whom 34 (64.15%) aspirated, and 5 (9.43%) developed aspiration pneumonia. None of the four risk factors (i.e. low LOC, poor oral hygiene, enteral nutrition, and dependent for oral intake) were significantly associated with aspiration pneumonia. The majority of participants (94.11%) had adequate oral hygiene, which was assessed subjectively (i.e. visual inspection of the oral cavity) by their treating SLT. All five participants who developed aspiration pneumonia were dependent for oral intake compared with those without aspiration pneumonia; however, this association was not significant (*p* = 0.040).

## Discussion

The majority of participants had co-occurring speech, language and swallowing diagnoses, of which dysphagia, aphasia, and dysarthria were the most frequent combination of conditions, which is supported by the literature (De Cock et al., 2020; Flowers et al., 2013; Stipancic et al., 2019). Co-occurring dysphagia, aphasia and dysarthria may have a challenging effect on intervention. Individuals with receptive aphasia might have difficulty understanding and following instructions for the management of other co-occurring conditions, which influences the selection of intervention techniques. Individuals who have expressive aphasia may have difficulty in communicating symptoms of dysphagia, which can lead to untreated aspiration, delayed intervention, and increased risk of aspiration pneumonia, which can be prevented (Lazar & Boehmer, 2017). Individuals with co-occurring speech and language difficulties may have difficulty in communicating effectively with family and friends, which can lead to frustration and depression (Rose et al., 2019). Additionally, communication difficulties will impact educating and counselling individuals, and affect their ability to make decisions on treatment options (McCormick et al., 2017). Individuals who have co-occurring dysphagia, aphasia, and dysarthria post-stroke have increased complexity that requires a wider range of SLT intervention (Mitchell et al., 2021). Three participants had co-occurring apraxia of speech, dysarthria, and dysphagia, which is an unusual finding, but can occur as reported in previous studies (Ghoreyshi et al., 2021; Trupe et al., 2018). Apraxia of speech and dysphagia may co-occur because of overlapping anatomy and similar motor programming functions for speech and swallowing (Trupe et al., 2018).

The majority of participants were referred to speech therapy within 48 h, which is not compliant with the World Stroke Organization (Feigin et al., 2022), which recommends SLT referrals and swallow screening occur within 24 h of stroke admission. In South Africa’s public sector, the average time from admission to SLT referral was 72 h (Balie et al., 2019), which was a longer delay in referral in comparison, and suggests that private hospitals may be timelier at referring individuals with stroke to SLT services. There was a trend towards delayed SLT referrals being associated with an increased LOS, which was a consistent finding in previous public sector studies (Balie et al., 2019; Han et al., 2018; Viljoen et al., 2014). Delayed SLT referrals are associated with delayed communication and swallowing intervention, which are related with subsequent consequences such as unsafe oral feeding, aspiration pneumonia and dehydration, and influences when one is medically stable for discharge (Bray et al., 2017; Han et al., 2018; Schwarz et al., 2018; Viljoen et al., 2014). In order to reduce LOS in the acute setting, SLTs need to raise awareness of the consequences of delayed referrals, establish referral guidelines and pathways for healthcare professionals who are first in contact with individuals with stroke, and monitor the efficiency of speech and swallow screening programmes (Bray et al., 2017; Glize et al., 2015).

Participants stayed in hospital longer (median LOS: 15 days) in the private sector, compared with the public sector (median LOS: 12.5 days) (Balie et al., 2019); however, this comparison should be read with caution as there is disparity between private and public healthcare and access to follow-up rehabilitation, which may have influenced one’s LOS in hospital and has implications on patient outcomes (Smythe et al., 2022). Although there were no significant associations between SLT conditions and LOS, participants with dysphagia and aphasia were associated with a longer LOS, which is in agreement with the literature (Bray et al., 2017; Lazar & Boehme, 2017; Mitchell et al., 2021). Participants with dysphagia who aspirated and developed aspiration pneumonia stayed in hospital longer compared with those who did not aspirate, which was similarly reported in previous studies (Finlayson et al., 2011; Schwarz et al., 2018). Dysphagia with aspiration and aspiration pneumonia may be associated with a longer LOS as a result of increased time in transitioning from enteral nutrition to total oral intake, poor medical status, and physical disability (Schwarz et al., 2018; Smithard, 2016). Timely dysphagia intervention is necessary to minimise aspiration and reduce the risk of aspiration pneumonia, which can improve patient outcomes and reduce healthcare costs and LOS (Bray et al., 2017; Han et al., 2018; Viljoen et al., 2014).

Instrumental swallow assessments (i.e. VFSS, FEES) were not used as frequently as CSE, which may have been because of limited medical aid insurance (Benatar, 2013), unsuitable candidates, especially those who had severe physical disability or poor medical status (Brodsky et al., 2019; Schwarz et al., 2018), as well as accessibility challenges for VFSS because of the effect of COVID-19 precautions (Miles et al., 2021). Instrumental studies are the gold standards for swallow assessments (Helliwell et al., 2023) and should be used frequently if possible and where appropriate, as they yield information about the presence or absence of aspiration, including silent aspiration, identify swallow function and impairment contributing to aspiration, and provide the opportunity to assess the efficacy of trial interventions (Seo et al., 2021). Although instrumental studies are valuable, CSE are inexpensive; they are reasonably accurate at assessing the risk of oropharyngeal aspiration, and are essential when VFSS and FEES are not available (Romano et al., 2014).

Participants received an average of 11 intervention sessions in a median of 15 days, in this study; whereas, individuals post-stroke in the public sector had an average of two intervention sessions in a median of 12.5 days (Balie et al., 2019). There were more SLT intervention sessions per median time (0.73) in the private sector, compared with the public sector (0.16). A high frequency of intervention in the acute setting is beneficial as it is associated with favourable outcomes such as earlier stroke recovery (Harvey et al., 2021), and improvement of swallowing ability (Smithard, 2016). An increased number of intervention sessions in the private sector could have occurred because of a lower patient demand and increased availability of SLTs (Pillay et al., 2020; Young, 2016). In addition, SLTs in the present study treated individuals on weekends in addition to weekdays, which was part of standard care, which may have contributed to a high number of intervention sessions.

Moderate to severe physical disability (mRS 3–6) post-stroke on admission and discharge was associated with dysphagia and severe physical disability (mRS 5) was associated with aspiration pneumonia at discharge. These findings are supported by previous studies (Langmore et al., 1998; Schwarz et al., 2018). Speech-language therapists should be aware of these associations, and incorporate this information into dysphagia management, such as advising the multidisciplinary stroke team to assist individuals post-stroke who have physical disability by positioning them upright for mealtimes and practising self-feeding, which will facilitate safe swallowing and reduce the risk of aspiration (Schwarz et al., 2018).

Dysphagia with aspiration was significantly associated with poor functional level of oral intake (i.e. altered consistency diets and enteral nutrition) at admission and discharge. Individuals post-stroke who have dysphagia with aspiration are at an increased risk of oral and pharyngeal phase difficulties; hence they are more likely to receive altered consistency diets to assist with safe eating and swallowing, and enteral nutrition to optimise nutrition and hydration (IDDSI, 2019; Smithard, 2016). Although dehydration was not a significant complication post-stroke in the private sector, research has found that dysphagia post-stroke can predispose individuals to dehydration because of fear of choking and poor oral intake because of reduced palatability of altered food or liquid consistencies, such as thickened liquids (Jeong et al., 2018).

The majority (62.5%) of participants who were placed on enteral nutrition following admission were still receiving enteral nutrition at discharge. This result indicated that there was limited progress in the mode of nutritional intake from admission to discharge. Long-term enteral nutrition may be associated with severe physical disability (mRS: 3–5), co-occurring speech, language and swallowing conditions, elderly and retired population, ischaemic stroke, and low LOC (Dziewas et al., 2004; Kenzaka et al., 2017; Schwarz et al., 2018; Viljoen et al., 2014). Caregivers need to be counselled that although enteral nutrition is important for maintaining nutrition and hydration, it does not prevent aspiration and aspiration pneumonia; therefore, precautionary measures are necessary such as positioning adjustments and regular oral care (Langdon et al., 2009; Mandell & Niederman, 2019).

Ten participants died during the study period of whom all had two to four co-occurring SLT diagnoses. Participants who had dysphagia were more likely to die compared with those without dysphagia, which was similarly reported in the public sector (De Villiers et al., 2011). Dysphagia post-stroke is associated with mortality because of increased risk of related complications such as enteral nutrition, aspiration pneumonia, dehydration, weight loss, and malnutrition (Feng et al., 2019; Jeong et al., 2018; Schwarz et al., 2018). From the five participants who developed aspiration pneumonia, 40% died during the study period. There were no significant associations between mortality and aspiration pneumonia, which may have occurred because of the low incidence of aspiration pneumonia. In comparison, previous studies with large study populations have reported associations between aspiration pneumonia and mortality post-stroke (Feng et al., 2019; Schwarz et al., 2018), and increased risk of mortality up to 1-year post-stroke (Teh et al., 2018). Early acute care SLT intervention is necessary to manage dysphagia post-stroke (Bray et al., 2017) and prevent complications, such as aspiration pneumonia, which are associated with increased risk of mortality (Feng et al., 2019).

The majority of participants who had dysphagia post-stroke experienced aspiration (64.15%), which is in alignment with the literature (Aoki et al., 2016; Mandell & Niederman, 2019; Martino et al., 2005). Most participants with dysphagia who aspirated did not develop aspiration pneumonia (85.29%). A low incidence of aspiration pneumonia compared with aspiration elucidates previous studies’ findings that not everyone who aspirates develops aspiration pneumonia, and additional risk factors are associated with the development of aspiration pneumonia (Langmore et al., 1998, 2002; Mandell & Niederman, 2019). None of the four risk factors, namely poor oral hygiene, dependent for oral intake, low LOC, and enteral nutrition, were significantly associated with aspiration pneumonia post-stroke. Previous studies have reported that regular oral care, dysphagia management, and medical intervention (e.g. daily oxygen therapy, antibiotics) are associated with reduced incidence of aspiration pneumonia (Mandell & Niederman, 2019; Murray & Scholten, 2018). Although not significant, all five participants who developed aspiration pneumonia in the present study, were dependent on oral intake. Research has shown that being dependent on oral intake is associated with aspiration pneumonia because of poorly trained caregivers and rushed feeding pace, resulting in aspiration (Huang et al., 2006; Langmore et al., 1998, 2002; Wright et al., 2008). In order to prevent aspiration and aspiration pneumonia, nursing staff and caregivers in acute care require training by an SLT on how to safely feed individuals with post-stroke dysphagia, who are dependent for being fed (Huang et al., 2006; Pierpoint & Pillay, 2020; Wright et al., 2008).

The study’s strengths included a prospective cohort design that was advantageous as it allowed for the ability to control and access information from participants’ hospital folders during data collection and analysis (Gregory & Radovinsky, 2012); multiple outcomes were investigated; and the temporal sequence between risk factors and associated outcomes was determined (De Rango, 2016). The study was set at five private hospitals in the Western Cape representing four hospital network organisations, which increased external validity.

There were several limitations that were encountered during the study’s data collection process, which may have affected the validity of results. Convenience sampling was used, which may have introduced bias and the lack of generalisability. The study’s sample size was small, which affected the statistical power of several findings.

Future recommendations include obtaining strong evidence-based data on speech, language and cognitive-communication disorders post-stroke, assessment tools, intervention, and association with patient outcomes; as well as obtaining more data on associations between risk factors and aspiration pneumonia post-stroke in South Africa.

## Conclusion

This prospective cohort study investigated associations between speech, language and swallowing conditions post-stroke and outcomes in the private acute care sector in the Cape Metropolitan area, South Africa. Co-occurring speech, language and swallowing conditions frequently occurred post-stroke. Participants who were referred to speech therapy later than 24 h, and who had dysphagia with aspiration and aspiration pneumonia, had a longer LOS. Dysphagia and aspiration pneumonia were significantly associated with moderate to severe physical disability. Dysphagia with aspiration was significantly associated with poor functional level of oral intake. Participants with dysphagia had a higher likelihood of mortality, compared with speech and language conditions. None of the four risk factors, namely, poor oral hygiene, low LOC, dependency on oral intake, enteral nutrition, were associated with aspiration pneumonia, but all participants who had aspiration pneumonia were dependent on oral intake. The study has shown that dysphagia post-stroke was strongly associated with unfavourable outcomes in the private sector, which has implications for the acute setting such as early SLT referrals (< 24 h) and multi-disciplinary stroke management. Given the lack of existing research in private healthcare, this study has provided knowledge on speech, language and swallowing conditions post-stroke and association with risk factors, nature of SLT services, and outcomes in South Africa’s private acute care sector.

## Appendix 1 – The Functional Oral Intake Scale

### Functional Oral Intake Scale^1^

Tube dependent (levels 1-3)

1. No oral intake
2. Tube dependent with minimal/inconsistent oral intake
3. Tube supplements with consistent oral intake

Total oral intake (levels 4-7)

4 Total oral intake of a single consistency
5 Total oral intake of multiple consistencies requiring special preparation
6 Total oral intake with no special preparation, but must avoid specific foods or liquid items
7 Total oral intake with no restrictions

## Appendix 2 – The Modified Rankin Scale

**Table:**

**MODIFIED**	**Patient Name:** _________________________
**RANKIN**	**Rater Name: _________________________**
**SCALE (MRS)**	**Date:__________________________**

**Table:**

Score	Description

0	No symptoms at all
1	No significant disability despite symptoms; able to carry out all usual duties and activities
2	Slight disability; unable to carry out all previous activities, but able to look after own affairs without assistance
3	Moderate disability; requiring some help, but able to walk without assistance
4	Moderately severe disability; unable to walk without assistance and unable to attend to own bodily needs without assistance
5	Severe disability; bedridden, incontinent and requiring constant nursing care and attention
6	Dead

**TOTAL (0–6):**_______

## Appendix 3 – Coding Manual

### Key

For all participants:

- 1 = For applicable or present characteristics
- 0 = For non-applicable/not present
- Solid red block = missing information/ unable to be deduced
- The number 1 to tally the number of incidences occurred (e.g. 1,1,1…Final total = 3)

### Demographic Information

- Patient Reference: ALIVE = A + number serially
- Age = Number in whole years on date of admission
- Language = Enter 1 or 0 under the respective language/combination of languages (e.g. English, English and Afrikaans) the participant speaks.
- Sex = Enter 1 or 0 under the respective sex (e.g. female, male) of the participant.
- Employment = Enter 1 or 0 under the respective employment status of the participant (e.g. employed, unemployed).

### Admission

- Date of admission (i.e. the day the patient was admitted to hospital for a stroke diagnosis) = DD/MM/YYYY (record on participant coding document)
- Level of physical ability at admission according to mRS (value of 0-6) – Consult with participants’ Physiotherapists. Modified Rankin Scale (mRS) value 0–6 = Record 1 under appropriate score; if not assigned by PT then assign based on PT notes. Enter the score (e.g. 6) under the column ‘mRS’ score.
  ■ 0 = no symptoms
  ■ 1 = no significant disability despite symptoms; able to carry out all usual duties and activities
  ■ 2 = slight disability; unable to carry out all previous activities, but able to look after own affairs without assistance
  ■ 3 = moderate disability; requires some help, but able to walk without assistance
  ■ 4 = moderately severe disability; unable to walk and attend to bodily needs without assistance
  ■ 5 = severe disability; bedridden, incontinent and requiring constant nursing care and attention
  ■ 6 = dead
- Mode of nutritional intake according to FOIS (refer to scale). FOIS value 1-7 = Record 1 under appropriate score, and enter the score (e.g. 4) under the column ‘FOIS’ score.
  ■ 1 = NPO
  ■ 2 = tube dependent, minimal attempts of food or liquid
  ■ 3 = tube dependent with consistent oral intake of food or liquid
  ■ 4 = oral diet; single consistency
  ■ 5 = oral diet; multiple consistencies; requiring special preparation or compensations
  ■ 6 = oral diet; multiple consistencies; no special preparation; specific food limitations
  ■ 7 = total oral diet with no restrictions
- Level of consciousness/GCS scale score: If GCS score is recorded, and if the score is above 12/15, then consider the patient alert. If the score is below 11/15, then consider the patient low level of consciousness.

### Discharge

- Date/Duration
  ■ Discharge date = DD/MM/YYYY (record on participant coding document).
  ■ Length of hospital stay (days) = Number – i.e. the number of NIGHTS between admission date and discharge date.
- Destination
  ■ Rehabilitation/Intermediate care facility – patient is going here primarily for rehabilitation (e.g. Life Rehab; Nurture Cape View).
  ■ Long-term care facility – patient is being placed here indefinitely for long-term nursing care (e.g. Oasis Care Centre).
- Status
  ■ Level of physical ability at discharge according to mRS (vale of 0-6) – Consult with participants’ Physiotherapists: Record 1 under appropriate score and enter the mRS score under the column ‘mRS Score’. Mode of nutritional intake according to FOIS (refer to scale): Record 1 under appropriate score, and enter the FOIS score under ‘FOIS score’.
  ■ Level of consciousness according to GCS: If GCS score is recorded, and if the score is above 12/15, then consider the patient alert. If the score is below 11/15, then consider the patient low level of consciousness.

### Stroke Details

- Ischaemic = infarct.
- Haemorrhagic = bleeding.
- Previous stroke = Any stroke that occurred prior to the stroke for which they were currently admitted for.
- Type of stroke
  ■ Unspecified – unknown whether the stroke is ischaemic or haemorrhagic (stated by the patient’s Doctor).
  ■ Record detailed information on type of stroke on participant coding document.
  ■ NIH Stroke Scale Score: Record 1 under appropriate score, and enter the
  ■ NIH score under the column ‘NIH score’.
    0 = No stroke.
    1-4 = Minor stroke.
    5-15 = Moderate stroke.
    16-20 = Moderate to severe stroke.
    21-42 = Severe stroke.
- Hemiparesis: Any weakness or slight paralysis on one side of the body.
- Hemiparalysis: Paralysis of one side of body.

### Comorbidities

- Incidences of new co-morbidities: List all new conditions which has been diagnosed during length of current hospitalisation – Record this on participant coding document.
- Other comorbidities – List any additional comorbidities on participant coding document.
- Cardiac: Check for specific cardiac conditions (e.g. Atrial Fibrillation (AF).
- Take note of the following abbreviations:
  ■ AF = Atrial Fibrillation
  ■ AKA = Above the Knee Amputation
  ■ ARF = Acute Renal (kidney) Failure
  ■ BKA = Below the Knee Amputation
  ■ BP = Blood Pressure
  ■ Ca = Cancer
  ■ CHD = Coronary Heart Disease (type of Hypertensive Heart Disease)
  ■ CHF/CCF = Congestive Heart Failure/ Congestive Cardiac Failure
  ■ CKD = Chronic Kidney Disease
  ■ COPD = Chronic Obstructive Pulmonary Disease
  ■ CVD = Cardiovascular Disease
    * CVD also includes Coronary Artery Diseases (CAD), such as angina
  ■ DM = Diabetes Mellitus
  ■ DNR = Do Not Resuscitate
  ■ DVT = Deep Vein Thrombosis
  ■ ESLD = Hepatic/ Liver Failure
  ■ ESRD = Kidney/ Renal Failure
  ■ GO/ERD = Gastroesophageal Reflux Disorder
  ■ HBP = High Blood Pressure/ Hypertension
  ■ HPT/ HPN = Hypertension
  ■ IBD = Inflammatory Bowel Disease
  ■ IBS = Irritable Bowel Syndrome
  ■ IDDM = Insulin-dependent diabetes mellitus/ Type 1 diabetes
  ■ IHD = Ischaemic Heart Disease
  ■ LVH = Left Ventricular Hypertrophy (type of Hypertensive Heart Disease)
  ■ MI = Myocardial Infarction
  ■ MNE = Motor Neuron Disease
  ■ PE = Pulmonary embolism (a type of blood clot in the lungs)
  ■ PVD = Peripheral Vascular Disease
  ■ RDS = Respiratory Distress Syndrome
  ■ SOB = Shortness of breath
  ■ UTI = Urinary tract infection
- Types of liver disease:
  ■ Nonalcoholic fatty liver disease
  ■ Hepatitis C
  ■ Hepatitis B
  ■ Cirrhosis of the liver
  ■ Alcoholic hepatitis
  ■ Hepatitis A
  ■ Hemochromatosis

### Dentition

- Oral hygiene status: Good/Clean = Tongue is clean with no white colouring; no food particles in between teeth/dentures/cheeks; mouth is cleaned with mouthwash/toothpaste and mouth kit (i.e. sponge stick, gauze, toothbrush).
- Oral hygiene status: Poor = Oral thrush (white colouring on tongue); food particles in between teeth/cheeks/palate; skin peeling off lips; mouth sores; no mouthwash/toothpaste or mouth kit.
- Tally the number of times oral hygiene was reported good/per total no. of sessions, and give the total number when patient is discharged. Does not include assessment session.
- Consider oral care “good” if reported or not reported in case notes. Consider oral care “poor” if reported in case notes.
- >50% (e.g. 6/10 sessions) of oral hygiene noted good/per total. no. of sessions = Consider routine oral care implemented.
- <50% (e.g. 3/10 sessions) of oral hygiene noted/per total no. of sessions = Consider lack of routine oral implemented.
- Enter 1 under “good” or “poor” depending on percentage (no. of times oral hygiene noted good/total no. of sessions).

#### Pneumonia (as verified by Radiologists’ interpretation of chest-X-rays and by the medical diagnosis made by the participants’ Physicians)

- Hospital-acquired pneumonia = Any pneumonia contracted by a person that is hospitalised (e.g. Klebsiella pneumonia).
- Aspiration pneumonia = Pneumonia caused as a result of aspiration of food or liquid.
- Community-acquired pneumonia = Any pneumonia contracted by a person outside of the hospital environment (i.e. before admitted to hospital).
  *Enter 1 or 0 under the respective pneumonia options.

### Dysphagia Interventions

- Mode of nutritional intake = Any modification to the method of eating or feeding a patient (e.g. slow pace, small bolus volumes, via syringe instead of straw etc.).
- Sensory intervention = Icing to facial musculature, different tastes (e.g. sour, cold), thermal tactile stimulation.
- Oral diet and oral supplements = The patient receives an oral diet (e.g. puree/soft/full) and also oral supplements (e.g. Fresubin drinks, Kcal crème etc.).
- Enteral nutrition and oral intake = The patient is tube-fed via Nasogastric Tube (NGT) or PEG tube, and also receives small volumes of food and/or liquid orally.

### Nature of SLT Services

- The number of dysphagia/aphasia/dysarthria/apraxia of speech sessions does not include the assessment session.
- Time from admission to SLT referral (hours) = Number (e.g. 24).
- Aphasia Rx: Consider word retrieval rx as part of expressive language rx.
- Total number of intervention sessions = Number of 15-minute interval therapy sessions (i.e. the number of therapy/contact time sessions we’ve had with the patient).
- If there are combined therapy sessions (e.g. dysphagia and aphasia rx in one session) then count as one session for each condition (1 session for dysphagia and 1 session for aphasia).
- Length of Intervention = The period of time the patient could have received therapy in hospital (i.e. excluding assessment session, count all days till discharge, irrespective of whether the patient was or wasn’t seen by Speech Therapy for therapy sessions).
- “Other” aphasia/apraxia of speech/dysarthria therapy content = Record which other therapy techniques were used in sessions, on participant coding document.
- Referrals = State the number of referrals by SLT. If referrals were made, state which Health Professionals were referred to (e.g. Dietitian, OT) on the participant coding document.
